# Effect of a Multicomponent Intervention on Acute Myocardial Infarction Diagnosis and Treatment in Tanzania: The MIMIC Implementation Trial

**DOI:** 10.21203/rs.3.rs-5599267/v1

**Published:** 2024-12-12

**Authors:** Julian Thornton Hertz, Joshua E Nworie, Frida M Shayo, Sophie W Galson, Lauren Coaxum, Ipyana Daniel, Patrick S Makambay, Ally M Akrabi, Gloria J Manyangu, Nathan M Thielman, Gerald S Bloomfield, Francis M Sakita

**Keywords:** acute myocardial infarction, sub-Saharan Africa, emergency department, evidence-based treatment, Tanzania

## Abstract

**Background:**

In Tanzania, acute myocardial infarction (AMI) is under-diagnosed, and uptake of evidence-based care is sub-optimal. Using an implementation science approach, an intervention was developed to address local barriers to care: the Multicomponent Intervention for Improving Myocardial Infarction Care in Tanzania (MIMIC).

**Methods:**

This single-arm pre-post trial was conducted in a northern Tanzanian emergency department (ED). During the pre-intervention phase (February–August 2023) and the post-intervention phase (September 2023–August 2024), adults presenting with chest pain and/or dyspnea were prospectively enrolled and their ED care was observed. AMI was defined by Fourth Universal Definition criteria. Telephone follow-ups were conducted to ascertain 30-day mortality. Pearson’s chi-squared was used to compare care before and after MIMIC implementation.

**Results:**

A total of 275 participants were enrolled in the pre-intervention phase and 577 were enrolled in the post-intervention phase. Following MIMIC implementation, significant increases were observed in ECG testing (89.4% of post-intervention participants vs 55.3% pre-intervention, OR 6.82, 95% CI: 4.79–9.79, *p*<0.001), troponin testing (78.0% of post-intervention participants vs 41.4% pre-intervention, OR 4.99, 95% CI: 3.67–6.83, *p*<0.001), and AMI case identification (24.4% of post-intervention participants vs 14.9% pre-intervention, OR 1.84, 95% CI: 1.26–2.73, *p*=0.002). Among participants with AMI, significant increases were observed in evidence-based treatment, including aspirin (71.6% among post-intervention AMI participants vs 34.4% pre-intervention, OR 4.80, 95% CI: 2.31–10.37, *p*<0.001), clopidogrel (65.2% among post-intervention AMI participants vs 26.8% pre-intervention, OR 5.03, 95% CI: 2.37–11.39, *p*<0.001), and heparin (43.2% among post-intervention participants vs 4.9% pre-intervention, OR 13.76, 95% CI: 3.99–93.79, *p*<0.001). Thirty-day survival among AMI participants did not change following MIMIC implementation (63.8% among post-intervention AMI participants vs 61.0% pre-intervention, OR 1.31, 95% CI: 0.54–2.31, *p*=0.739).

**Conclusions:**

The MIMIC intervention was associated with large increases in uptake of AMI testing, case identification, and evidence-based treatment in a Tanzanian ED.

## Introduction

Acute myocardial infarction (AMI) is the leading cause of death worldwide, accounting for approximately 3 million deaths globally each year.(1)In recent decades, substantial reductions in AMI mortality have been achieved in high-income countries through widespread adoption of evidence-based care, including broad screening for AMI in emergency departments (EDs) and early administration of aspirin and other evidence-based therapies.(2, 3, 4, 5) Because aspirin is both inexpensive and effective, the World Health Organization (WHO) has prioritized the use of aspirin in AMI, highlighting it as a “best buy” for reducing noncommunicable disease mortality and morbidity.(6) Although AMI is common globally, there has been much less study of AMI in sub-Saharan Africa (SSA).(7, 8, 9) Two recent systematic reviews found that there had been no studies investigating strategies to increase uptake of aspirin therapy among AMI patients in all of SSA and no published quality improvement interventions for AMI care in SSA in general. (10, 11)

A growing body of evidence suggests that AMI outcomes in SSA are poor. In Tanzania, for example, recent studies found that few emergency department (ED) patients with possible AMI symptoms underwent AMI testing, resulting in about 90% of AMI cases being misdiagnosed.(12, 13) Further studies revealed that less than one in four AMI patients received aspirin or other evidence-based therapies and very few AMI survivors were taking aspirin at 30 day follow-up, resulting in an alarmingly high thirty-day mortality rate of 43%.(12, 14, 15) As there were no published interventions designed to improve AMI care in sub-Saharan Africa,(9) our team developed a contextualized quality improvement intervention using an implementation science approach.(16) The intervention, Multicomponent Intervention for Improving Myocardial Infarction Care (MIMIC), is the first published quality improvement intervention for AMI care in Sub-Saharan Africa.(17)

To evaluate the effect of the MIMIC intervention on AMI care, we conducted a single-arm pilot trial of the MIMIC intervention in a Tanzanian ED. In this trial, we compared AMI diagnostic and care metrics from before and after implementation of the MIMC intervention to estimate effects on AMI testing, treatment, and outcomes in the first trial of an intervention for improving uptake of evidence-based AMI care in SSA. These findings will inform implementation and scale-up of similar quality improvement work for AMI care in other resource limited settings.

## Methods

### Setting

This study took place at Kilimanjaro Christian Medical Centre (KCMC) in Moshi, Tanzania. KCMC is a tertiary care and zonal hospital serving approximately 15 million people. Although KCMC lacks a cardiologist and the capacity for percutaneous intervention or cardiac surgery, it is well stocked with basic AMI medications, which includes aspirin, clopidogrel, nitrates, beta blockers, thrombolytics, statins, heparin, and other antihypertensives. The hospital also has AMI diagnostic tools such as electrocardiography (ECG) machines, echocardiograms, and both point-of-care and laboratory-based troponin assays. The KCMC ED is staffed 24 hours per day by physicians, some of whom are residency trained in emergency medicine.

### Study Design and Timeline

This single-arm pilot trial was conducted for one year, from 1 September 2023 to 31 August 2024. The full study protocol for this trial has been previously published.(18) Baseline pre-intervention comparative data was collected for seven months prior to the implementation of the MIMIC intervention, from 1 February 2023 to 31 August 2023. Observational data from the pre-interventional phase have been submitted for publication separately (publication pending).

### Pre-Intervention Period

During the pre-intervention study period, trained research assistants screened all patients presenting to the KCMC ED. Any patient aged ^3^18 years presenting with chest pain or shortness of breath was eligible for inclusion. Patients with self-reported fever and patients whose chest pain was secondary to trauma were excluded. Patients were consecutively screened by the research team from 8AM until 11PM seven days per week, and all eligible patients were invited to participate in the study. Written informed consent was obtained from all participants prior to enrollment. After enrollment, research assistants administered a brief questionnaire to participants to elicit information about medical history and presenting symptoms. Research assistants directly observed participants’ ED care and collected information regarding AMI testing and treatment directly from the electronic medical record. Specifically, the research team collected information about whether any ECG or cardiac biomarker testing was ordered, all treatments administered, the results of all laboratory testing, and final documented diagnoses. Digital images of all ECG tracings were also collected by the research team.

### MIMIC implementation

The MIMIC intervention started on September 1st, 2023. This quality improvement initiative was executed by the KCMC ED clinical staff exclusively. The MIMIC intervention, along with the implementation mapping approach used to develop it, has been described in detail elsewhere.(16, 17) Briefly, MIMIC consists of five components: (1) unique “AMI suspect” triage cards placed by triage nurses on the stretchers of patients with chest pain or dyspnea, (2) an online training module reviewing evidence-based AMI diagnosis and care for all ED staff, (3) pocket cards summarizing AMI care that were distributed to all ED staff, (4) educational pamphlets for patients with AMI, and (5) a designated physician and nurse champion who were responsible for auditing AMI care and implementing all components of the MIMIC intervention. Because of the nature of the MIMIC intervention, participants were not blinded to their assignment to the pre-intervention phase or the post-intervention phase.

### Post-Intervention Participant Selection

During the post-intervention phase (September 1^st^, 2023 – August 31^st^, 2024), study procedures were identical to the pre-intervention phase, as described above. Trained research assistants screened all patients presenting to the KCMC ED, and enrolled adults with chest pain or shortness of breath. Enrollment was conducted from 8AM until 11PM seven days per week. Written informed consent was obtained from all participants prior to enrollment.

### Study Procedures

As during the pre-intervention phase, participants completed a brief questionnaire regarding medical history and presenting symptoms, and research assistants collected information regarding AMI testing, treatment, and diagnoses directly from the electronic medical record.

### Follow-up

During both the pre-intervention and post-intervention phases, participants were contacted via telephone 30 days after initial enrollment. At this time, a brief follow-up questionnaire was administered by the research team to assess vital status. If a participant could not be reached by telephone, a study team member visited their home to conduct the follow-up interview in person.

### ECG Interpretation

ECGs underwent external adjudication for study purposes. Two independent adjudicators reviewed the ECGs to determine if they met criteria for ST-elevation myocardial infarction (STEMI) as per Fourth Universal Definition of Myocardial Infarction Guidelines.(19) Adjudicators were ED physicians from the United States and Tanzania who had completed residency training in Emergency Medicine. A third adjudicator served as the tiebreaker in cases of disagreement.

### AMI Study Definition

AMI was defined in accordance with the Fourth Universal Definition of Myocardial Infarction guidelines. (19) Any participant meeting any of the following criteria was considered to have AMI: (1) ECG meeting STEMI criteria, (2) abnormally elevated troponin >99^th^ percentile upper reference limit with repeat three-hour troponin >11% higher or lower than the initial value, (3) abnormally elevated troponin >99^th^ percentile upper reference limit without advanced renal dysfunction (estimated glomerular filtration rate estimated glomerular filtration rate >15 ml/min/1.73m^2^) if only a single troponin assay was performed, and (4) final hospital discharge diagnosis of acute myocardial infarction.

### Other Study Definitions

History of tobacco use and alcohol use were defined by participant self-report. Baseline comorbidities, including hypertension, diabetes, prior stroke, and prior myocardial infarction were also defined by participant self-report.

### Outcomes

To estimate possible effect sizes of the MIMIC intervention on clinical outcomes, 11 key outcomes were evaluated: (1) the proportion of participants undergoing ECG testing in the ED, (2) the proportion of participants undergoing troponin testing in the ED, (3) the proportion of participants undergoing both troponin and ECG testing in the ED, (4) the proportion of participants identified with AMI per the study definition, (5) the proportion of participants meeting AMI criteria who were treated with aspirin in the ED, (6) the proportion of participants with AMI treated with clopidogrel or other P2Y12 inhibitor in the ED, (7) the proportion of AMI participants treated with heparin in the ED, (8) the proportion of AMI participants treated with a statin in the ED, (9) the proportion of participants with AMI treated with a thrombolytic, (10) the proportion of AMI participants receiving a referral to a specialty cardiac center from the ED, and (11) the proportion of participants with AMI alive at 30 days.

### Statistical Analyses

All statistical analyses were performed in the R Suite. Standard deviations were calculated for all continuous variables. Baseline characteristics of participants in the pre- and post-intervention period were compared via Pearson’s chi-squared (for categorical variables) or Welch’s t-test (for continuous variables). Fisher’s exact test was used when expected cell count was less than 5. Estimated glomerular filtration rate was calculated from serum creatinine level using the updated race-neutral CKD-EPI equation.(20) The proportion of participants who received each of the 11 key performance metrics was compared among pre- and post-intervention participants using Pearson’s chi-squared. Odds ratios and 95% confidence intervals were constructed directly from contingency tables. A threshold of <5% was used for statistical significance. No *a priori* assumptions were made about effect size; therefore, the post-intervention phase of the pilot trial was conducted for one year without a pre-specified target sample size.

### Patient and Public Involvement

Patients from northern Tanzania were involved in the design of the MIMIC intervention. As described elsewhere,(17) patients with recent AMI participated in the Design Team process, providing essential input on the design and refinement of the intervention

### Ethics approval and consent to participate

The study received ethical approval from the Tanzania National Institute for Medical Research (NIMR/HQ/R.8a/Vol. IX/2436), the Kilimanjaro Christian Medical Centre (Proposal 893), and Duke Health (Pro00090902). This trial was registered on clinicaltrials.gov (NCT04563546) on September 24^th^, 2020. All participants provided written informed consent prior to enrolment. This study conformed to the principles of the Helsinki Declaration.

## Results

**Figure 1:**
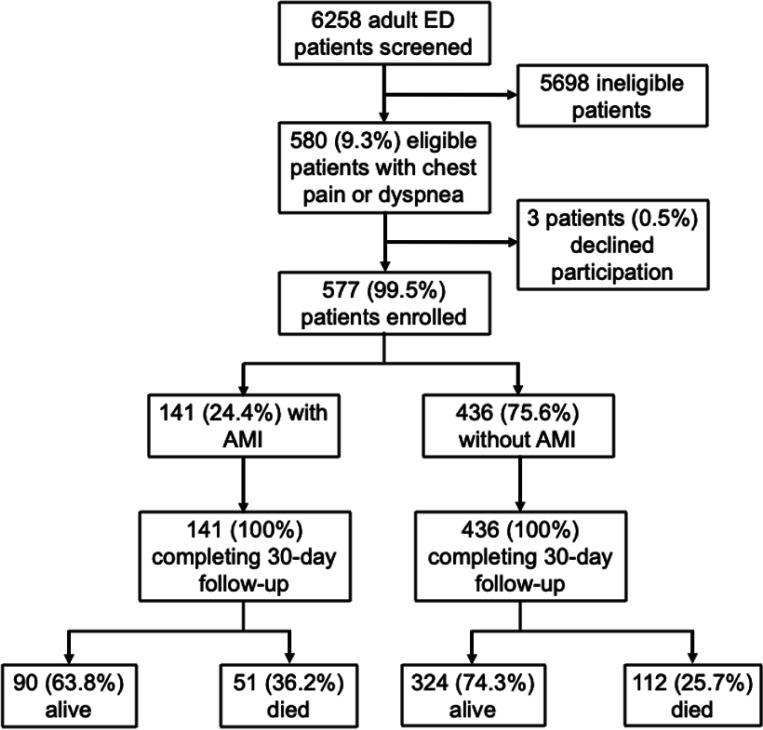
Flow chart of post-intervention participants

During the post-intervention study period, a total of 6258 adult patients presenting to the KCMC ED were screened, of whom 580 (9.3%) had chest pain or dyspnea and were eligible for enrollment. Three (0.5%) eligible participants declined to participate, and the remaining 577 (99.5%) provided informed consent and were enrolled (Fig 1). A total of 275 participants were enrolled during the pre-intervention period. Table 1 presents the baseline characteristics of the pre-intervention and post-intervention participants. Slightly more than half of participants were female (59.3% of post-intervention participants vs. 52.7% of pre-intervention participants, OR 0.77, 95% CI: 0.57–1.02, *p* = 0.071). The mean (sd) age of pre-intervention participants was 61.2 (19.6) years, compared to 62.4 (18.0) years for post-intervention participants (*p* = 0.416). There were otherwise no significant differences in baseline characteristics between pre-intervention participants and post-intervention participants, including tobacco use, history of hypertension, history of prior myocardial infarction, and symptom duration prior to ED presentation (Table 1).

Table 2 compares the uptake of diagnostic testing and AMI case detection during the pre-intervention and post-intervention periods. Compared to the pre-intervention period, uptake of ECG testing was significantly higher in the post-intervention period: 55.3% of pre-intervention participants received an ECG while 89.4% of post-intervention participants received an ECG (OR 6.82, 95% CI: 4.79–9.79, *p*<0.001). Similarly, uptake of troponin testing was significantly higher in the post-intervention period (78.0% vs 41.4%, OR 4.99, 95% CI: 3.67–6.83, *p*<0.001). In the setting of increased ECG and troponin testing, a significant increase in AMI case identification was observed: 24.4% of post-intervention participants met the study definition for AMI, compared to 14.9% of pre-intervention participants (OR 1.84, 95% CI: 1.26–2.73, *p*<0.001).

Table 3 presents the ED care observed among the 41 pre-intervention participants with AMI and 141 post-intervention participants with AMI. Uptake of aspirin therapy increased significantly following the implementation of the MIMIC intervention: 71.6% of post-intervention participants with AMI received aspirin in the ED, compared to 34.4% of pre-intervention participants with AMI (OR 4.80, 95% CI: 2.31–10.37, *p*<0.001). Similarly, uptake of clopidogrel, heparin, and statin therapy all significantly increased in the post-intervention period (Table 3). Although there was a trend towards increased uptake of thrombolytic therapy and increased referrals to cardiac centers in the post-intervention period, these differences were not statistically significant.

At thirty days, 1 pre-intervention participant who did not have AMI was lost to follow-up, but otherwise thirty-day follow-up was achieved for all other participants (overall follow-up rate 99.9%). At 30-day follow-up, 90 (63.8%) of the post-intervention participants with AMI were alive, as compared to 25 (61.0%) of the pre-intervention participants with AMI (OR 1.31, 95% CI: 0.54–2.31, *p*=0.739).

**Figure 2:**
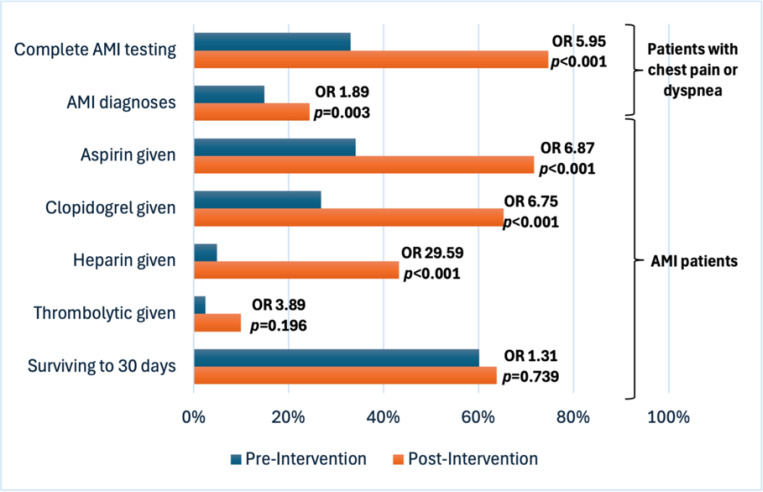
Effect of the MIMIC intervention on AMI diagnosis and care in the KCMC ED, 2023–2024 (N=832)

Figure 2 summarizes the overall impact of the MIMIC intervention on AMI diagnosis and care.

Figure 2. Effect of the MIMIC intervention on AMI diagnosis and care in the KCMC ED, 2023–2024 (N=832)

## Discussion

To our knowledge, this is the first study of an intervention to improve uptake of evidence-based AMI care in SSA. In a single-site study using a quasi-experimental pre-post design, we found that implementation of the MIMIC intervention was associated with large and statistically significant increases in AMI testing, diagnosis, and evidence-based treatment. Further evaluation in a multisite cluster-randomized trial is needed to evaluate the effect of MIMIC on AMI care and outcomes across Tanzania.

We found that implementation of MIMIC was associated with substantial increases in uptake of ECG screening and troponin testing. Importantly, this increase in testing uptake also resulted in increased AMI case identification. Prior studies have suggested that under-diagnosis of AMI is common in SSA,(21, 22) and the reasons for such under-detection are likely myriad.(22, 23) Although limited access to ECG and troponin testing is certainly an important reason for AMI under-diagnosis in some settings in SSA,(23) our prior research in Tanzania found that use of these diagnostic tests was poor even in EDs where they were available.(13) Subsequent qualitative studies found that ED providers in Tanzania often did not order such tests either because they did not feel comfortable interpreting such tests or because they failed to consider the diagnosis of AMI entirely.(16, 23) The MIMIC intervention employed strategies specifically designed to overcome these barriers, by providing additional training in AMI diagnosis and with use of special triage cards to prompt the treating physician to consider the diagnosis of AMI.(16, 17) At KCMC, the implementation of MIMIC resulted in substantial increases in AMI testing and diagnosis, an encouraging finding which suggests that tailored implementation strategies may substantially improve AMI case identification elsewhere in SSA.

We also observed large increases in uptake of evidence-based AMI therapies following implementation of the MIMIC intervention at KCMC. Specifically, uptake of aspirin, clopidogrel, and heparin increased by more than two-fold in the post-intervention phase. Although we also observed a trend in increased administration of thrombolytics and referrals to percutaneous coronary intervention (PCI)-capable facilities, these differences did not reach the threshold of statistical significance, likely due to small sample size. Although the WHO has highlighted aspirin treatment for AMI as a “best buy” for reducing global morbidity and mortality,(24) there have been no prior studies of interventions to improve uptake of aspirin in SSA.(10, 11) The large effect sizes observed in this single-center study are encouraging, and suggest that MIMIC may an effective strategy for improving evidence-based AMI therapy in similar settings across SSA. Notably, although the proportion of AMI patients receiving aspirin and evidence-based therapies did improve substantially in the post-intervention phase, nearly 30% of participants with AMI still did not receive aspirin. Thus, additional study is needed to understand the reasons for persistently sub-optimal uptake of these therapies and to develop additional strategies to further improve uptake.

Despite increases in AMI testing and evidence-based treatment, we did not observe a significant decrease in thirty-day mortality following implementation of MIMIC in our study. Our study was likely under-powered to detect differences in mortality; for example, the number needed to treat (NNT) to prevent-short term death in AMI for aspirin is approximately 42.(25) Thus, a much larger study would presumably be needed to detect mortality benefits from increased ED-based administration of aspirin, clopidogrel, and thrombolytics. Additional factors likely contribute to high AMI mortality rates in our setting beyond this ED-focused intervention. These include under-resourced primary care systems, which lead to poor management of comorbidities, limited emergency transport, delayed patient presentation, and significant distances to PCI-capable centers.(23) On average, participants presented after 4 days, and the closest PCI-equipped hospital is over 8 hours away by ground transport—constraints that reduce the effectiveness of time-sensitive reperfusion therapies.(26) Further efforts are needed improve AMI care and outcomes across the continuum of AMI care in Tanzania, including community education to reduce delays in hospital presentation,(27, 28) inpatient quality improvement activities to improve uptake of appropriate secondary preventative therapies at hospital discharge,(29, 30) and expansion of access to PCI across the country. There have been very few studies of interventions to improve AMI care outside of high-income countries. Two of the largest studies to date are the ACS QUIK trial in India(31) and the BRIDGE-ACS trial in Brazil.(32) In both of these studies, which were conducted in more resource-replete settings than our study setting, quality improvement interventions resulted in increases in uptake of evidence-based care, but also did not result in significant reductions in mortality.

This study had several strengths including use of a rigorous, guideline-based AMI definition(19) and robust follow-up procedures resulting in no AMI patient being lost to follow-up. This study also had several important limitations. First, this was a single-center study, so the generalizability of our findings to other settings in Tanzania or SSA is unknown. Second, like all studies using a longitudinal pre-post design, our results may have been confounded by unmeasured time-related variables. If there were background changes in AMI care unrelated to the MIMIC intervention occurring at KCMC during the study period, this would have impacted our estimates of effect sizes, but our group identified no secular trends in AMI care in Tanzania during this time. Finally, although we used standard guidelines for defining AMI,(19) we did not have access to coronary angiography or echocardiography in this study, which would have allowed us to confirm coronary atherothrombosis and wall motion abnormalities.

## Conclusions

In conclusion, in this single center study, implementation of a tailored intervention for improving AMI care was associated with substantial increases in AMI diagnoses and evidence-based care, but was not associated with reduced mortality. Use of an implementation science approach, which allowed for contextual tailoring of the intervention to address local barriers to care, likely contributed to the effectiveness of the MIMIC intervention in this setting. Additional studies are needed to evaluate the effectiveness of MIMIC in sites across Tanzania, and further research is needed to develop interventions to reduce AMI mortality across SSA.

## Figures and Tables

**Table 1.** Baseline characteristics of adult patients presenting to the KCMC ED with chest pain or shortness of breath, 2023–2024 (N=832)

	Post-intervention participants (N=577)		Pre-intervention participants (N=275)		Odds ratio (95% CI)	*p*
Characteristic	n	(%)	n	(%)		
Sex						
Female	304	(52.7%)	163	(59.3%)		
Male	273	(47.3%)	112	(40.7%)	0.77 (0.57–1.02)	0.071
History of tobacco use	202	(35.0%)	95	(34.5%)	0.98 (0.72–1.32)	0.894
History of alcohol use	414	(71.8%)	201	(73.1%)	1.07 (0.78–1.48)	0.683
History of hypertension	373	(64.6%)	164	(59.6%)	0.81 (0.60–1.09)	0.157
History of diabetes	158	(27.4%)	63	(22.9%)	0.79 (0.56–1.10)	0.164
History of heart failure	137	(23.7%)	73	(26.5%)	1.16 (0.83–1.61)	0.375
History of prior MI	36	(6.2%)	11	(4.0%)	0.63 (0.30–1.23)	0.181
History of stroke	26	(4.5%)	10	(3.6%)	0.81 (0.36–1.66)	0.555
History of HIV	19	(3.3%)	17	(6.2%)	1.94 (0.98–3.81)	0.051
	Post-intervention participants (N=577)		Pre-intervention participants (N=275)			*p*
	mean	(sd)	mean	(sd)		
Age (years)	62.4	(18.0)	61.2	(19.6)		0.416
Symptom duration prior to ED presentation (days)	4.9	(11.8)	4.2	(5.8)		0.260
Systolic blood pressure (mmHg)	141.8	(32.6)	141.7	(36.1)		0.964
Diastolic blood pressure (mmHg)	82.5	(20.1)	82.5	(20.9)		0.978

**Table 2.** Uptake of diagnostic testing and AMI case detection among adult patients presenting to the KCMC ED with chest pain or shortness of breath, 2023–2024 (N=832)

	Post-intervention participants (N=577)		Pre-intervention participants (N=275)		Odds ratio (95% CI)	*p*
	n	(%)	n	(%)		
ECG obtained	516	(89.4%)	152	(55.3%)	6.82 (4.79–9.79)	<0.001*
Troponin obtained	450	(78.0%)	114	(41.4%)	4.99 (3.67–6.83)	<0.001*
Both ECG and troponin obtained	431	(74.7%)	91	(33.1%)	5.95 (4.36–8.17)	<0.001*
AMI cases identified^a^	141	(24.4%)	41	(14.9%)	1.84 (1.26–2.73)	0.002*

a AMI case identification defined by participants meeting the study definition for AMI

* *p* < 0.05

**Table 3.** Uptake of evidence-based AMI therapy before and after implementation of the MIMIC intervention in a Tanzanian emergency department, 2023–2024 (N=832)

	Post-intervention AMI participants (N=141)		Pre-intervention AMI participants (N=41)		Odds ratio (95% CI)	*p*
Therapy	n	(%)	n	(%)		
Aspirin	101	(71.6%)	14	(34.4%)	4.80 (2.31–10.37)	<0.001*
Clopidogrel	92	(65.2%)	11	(26.8%)	5.03 (2.37–11.39)	<0.001*
Heparin	61	(43.2%)	2	(4.9%)	13.76 (3.99–93.79)	<0.001*
Statin	66	(46.8%)	10	(24.4%)	2.69 (1.26–6.21)	0.010*
Thrombolytic	14	(9.9%)	1	(2.4%)	3.89 (0.74–96.47)	0.196
Referral to cardiac center	19	(13.5%)	2	(4.9%)	2.84 (0.77–19.98)	0.169

* *p* < 0.05

## Data Availability

The datasets used and/or analyzed during the current study are available from the corresponding author on reasonable request.

## What is already known on this topic

- Research has shown that uptake of evidence-based care for acute myocardial infarction (AMI) is sub-optimal in Tanzania, but proven interventions for improving uptake of evidence-based AMI care are lacking in sub-Saharan Africa.
- **What this study adds** – This was the first trial of a tailored intervention for AMI care, developed using implementation mapping. In a Tanzanian hospital, we found that implementation of the MIMIC intervention resulted in large increases in AMI testing, AMI case identification, and evidence-based treatment.
- **How this study might affect research, practice or policy** – The MIMIC intervention should be considered for adaptation and scale-up across Tanzania and similar settings in sub-Saharan Africa. Larger multisite trials should be conducted to evaluate the effect of the MIMIC intervention on mortality and morbidity.

## Abbreviations

AMI: acute myocardial infarction
ECG: electrocardiogram
ED: emergency department
KCMC: Kilimanjaro Christian Medical Centre
MIMIC: Multicomponent Intervention for Improving Myocardial Infarction Care
PCI: Percutaneous coronary intervention
SSA: sub-Saharan Africa
STEMI: ST-elevation myocardial infarction
WHO: World Health Organization
